# The genomic mutation spectrums of breast fibroadenomas in Chinese population by whole exome sequencing analysis

**DOI:** 10.1002/cam4.2081

**Published:** 2019-03-09

**Authors:** Shang‐Nao Xie, Yuan‐Jie Cai, Bo Ma, Yanting Xu, Peng Qian, Juan‐Di Zhou, Fu‐Guang Zhao, Jie Chen

**Affiliations:** ^1^ Department of Breast Surgery Zhejiang Hospital Hangzhou P.R. China; ^2^ Division of Molecular Genetics Joingenome Diagnostics Hangzhou P.R. China; ^3^ Department of Bioinformatics Joingenome Diagnostics Hangzhou P.R. China

**Keywords:** copy number variation, fibroadenoma, germline mutation, *MED12*, somatic mutation, tumor mutational burden

## Abstract

Fibroadenomas (FAs) are the most common fibroepithelial lesions and the most common benign tumors of the breast in women of reproductive age. Although *MED12* mutations, an overwhelming majority of all mutations, and some other gene mutations have been found in FAs, the genomic landscapes of FAs are still not completely clear and the genomic mutation spectrums of FAs in Chinese population remains unknown. Here, by performing whole exome sequencing of 12 FAs and the corresponding normal breast tissues in Chinese Han population, we observed the somatic and germline landscapes of genetic alterations. We identified 16 recurrently mutated genes with 37 nonsynonymous or frameshift somatic mutations and 27 recurrent somatic copy number variants (CNVs). In these mutated genes, *MED12* was the most common in FAs, harboring 6 nonsynonymous/frameshift somatic mutations and 1 CNV. In addition, 6 germline mutations of tumor susceptibility genes in 5 FAs were identified and the tumor mutational burden of the 5 FAs was significantly higher than the other 7 FAs without germline mutations. This study provides genomic mutation spectrums of FAs in Chinese population and expand the genetic spectrum of FAs.

## 1 INTRODUCTION

Fibroadenomas (FAs) are the most common fibroepithelial lesions and the most common benign tumors of the breast. The incidence of FAs reported by Coriaty Nelson et al according to a study of 265,402 women in China is 241 per 100,000 among women under 35 years of age and 165 per 100,000 among women aged 35‐39 years.^1^ Although they are benign in essence, the risk of invasive breast cancer was 2.17 times higher among patients with FAs than that among the controls.^2^

FAs are often small but may be large and of rapid growth, especially juvenile FAs, which can raise clinical concern for phyllodes tumor. Previous studies suggest that there is a relationship between phyllodes tumors (PTs) and FAs.^3^, ^4^ Phyllodes include benign, borderline, and malignant tumors. The malignant phyllodes tumor may develop distant metastasis. Clinically, to differentiate FAs from phyllodes tumor is difficult. The difficulty exists in distinguishing some cellular FAs from PTs even for pathologists who specialize in breast pathology.^6^ The diagnosis of FAs is typically achieved by biopsy, and patients with larger lesions are often subjected to surgery. These procedures can be costly, produce anxiety and, in some cases, procedure‐related complications.^7^

Pathogenesis of FA remains unknown. Some studies have suggested a role for hormonal influence in FA development and growth.^8^, ^9^ Multiple and/or bilateral FAs have been associated with a positive family history.^13^ Previous targeted mutational screens of *TP53 *in FAs remain controversial.^14^, ^15^ A single *PIK3CA* mutation has also been reported from a screen of 10 FA tumors.^16^ Knowledge surrounding FA genetics and genomics has progressed rapidly over the last 20 years. Recent studies revealed that mediator complex subunit 12 (*MED12) *somatic mutations occurred frequently in fibroepithelial lesions, which were found in up to 65% of FAs.^17^, ^18^ Targeted deep sequencing of 21 FAs revealed mutations in the retinoic acid receptor alpha (RARA) gene in 3 (14.3%) of these cases, and missense mutations were also found in *FLNA*(1case), *PCLO*(1case), *CHD8*(1case), and *ROS1*(1case).^19^ Lozada et al reported myxoid FAs are different from conventional FAs, which lack *MED12 *mutations.^24^ These researches suggest that FAs may be heterogeneous and the genomic alterations of FAs may be not completely clear. Besides, there is no research studying the genomic mutation spectrums of FAs in Chinese population.

In order to identify whether the genomic mutations in FAs of the Chinese Han population is different from that of the other race, we conduct this study by means of whole exome sequencing of 12 matched FA tumors and corresponding normal tissues. We observed 16 recurrent genes with somatic mutations in 12 FAs and *MED12* harbored more somatic mutations than the other genes. In addition, 27 somatic copy number variants (CNVs) were identified. We also observed 6 germline mutations in 5 cases, and the tumor mutational burden (TMB) of these cases was higher than those with no germline mutation.

## 2 MATERIALS AND METHODS

### 2.1 Cases and subjects

Twelve fresh‐frozen FA tissues and the controls of normal breast tissue were obtained from 12 surgical patients from the Department of Breast Surgery, Zhejiang Hospital, Hangzhou. Clinicopathological characteristics of these tumors are shown in Table S1. Histological diagnosis of all tumors were confirmed by 2 pathologists. This study was approved by the Research Ethics committee of Zhejiang Hospital and informed consents were obtained from the patients.

### 2.2 DNA extractions

Fresh‐frozen tumor specimens were evaluated histologically to assess tumor cellularity when it was available. DNA was extracted from tumor specimens using Qiagen DNeasy Blood & Tissue Kit (Qiagen, Hilden, North Rhine‐Westphalia, Germany). The quality of DNA was assessed using Life Qubit Fluorometer 3.0 (Life Technologies, Grand Island, NY) according to the manufacturer’s protocol.

### 2.3 Library preparation and sequencing

For whole‐exome sequencing, DNA Library construction and hybrid selection of gDNA was performed using the Agilent SureSelect XT Reagent kit and SureSelect XT Human All Exon V6 (Agilent Technologies, Santa Clara, CA) with 2 μg of DNA input. Shear the gDNA by Covaris M220 (Thermofisher, MA) at fragment size 150‐200 bp before library preparation and purify the sample using Beckman Coulter AMPure XP beads (Beckman, MA) during the process. The quality of DNA Library was assessed using Life Qubit Fluorometer 3.0 and Agilent 2200 TapeStation Instrument (Agilent Technologies, Santa Clara, CA) respectively. Sequencing was performed on the Illumina HiSeq X‐ten (Illumina, CA) in high‐output mode with 150 bp paired‐end reads. Nine libraries were pooled in 1 lane. The mean Q20 was 95.49%, the mean Q30 was 89.82%, and the mean coverage was 227×.

### 2.4 Bioinformatics analyses

The bioinformatic analysis pipeline of the 12 samples is shown in Figure S1. Initially, the next generation sequencing (NGS) reads were submitted to the quality control software and filtered the low‐quality (<20) reads. After that the reads were mapped to the reference genome hg19 (GRCh37.p13) using the tool BWA.^25^ The picard toolkit (http://broadinstitute.github.io/picard) was used to sort the bam file and exclude duplicates. Base quality score recalibration and the local realignment around indels were performed using GATK.^26^ The GATK4 MuTect2 was used for somatic SNV/INDEL calling and the Varscan2 was used for germline SNV/INDEL calling.^27^, ^28^ The variants were filtered by at least 20X depth and 1% allele frequencies. Then the variants were annotated by SnpEff and Annovar with databases COSMIC, ICGC21, dbNSFP, Clinvar, OMIM.^29^, ^30^ The gene function step selected the variants in exon region, and retained the missense variants, the loss of function (LoF) variants, and the splice variants with ±2. The effect of SNV on the protein function was estimated by SIFT and PolyPhen2.^31^, ^32^ The missense variants was retained with the SIFT value of D and PolyPhen2 value of D/P. Then all the variants were filtered by the 1% mutation frequencies in 1000 Genomes and ExAC projects. Finally, we retained the last annotation file for the next analyses. The germline mutations were from the adjacent normal tissues, and the frequency of variants was between 40% and 60%. The somatic mutations were obtained by comparing DNA sequences between tumor tissues and the adjacent normal tissues.

In the somatic variant selection progress, we first filtered variants that were regarded as benign, like benign tumors in Intervar and Clinvar database. Second we filtered the variants that had rs ID in dbSNP (147) database but did not have records in COSMIC, ICGC21, and the Clinvar database. We further filtered the missense variants that did not have records in COSMIC, ICGC21, Clinvar database and had hits above 3 in the genome. Finally, we obtained 137 missense variants and 198 LoF variants that may have significant roles in FAs.

In the germline variant selection progress, firstly we filtered the variants that were regarded as benign, like benign tumors in Intervar and Clinvar database. Then we filtered the variants that had rs ID in dbSNP (147) database but did not have records in COSMIC, ICGC21, Clinvar database. Next we filtered missense variants that do not have records in COSMIC, ICGC21, Clinvar database and had above 3 hits in the genome. Finally, we filtered variants that were not among the 96 tumor susceptibility genes and got 6 germline high‐risk variants.

In the somatic CNV selection progress, we used the varscan2 CNV module and exome CNV to call the CNVs, and got the shared region where the CNVs were from.^33^ Next we retained the CNVs with a size >25 KB and selected the CNVs that contained the oncogenes and *MED12* gene. Then we filtered the CNVs that had records in DGV and retained the CNVs that were annotated as likely pathogenic or pathogenic in Clingen database, specifically retained the CNVs containing *MED12* gene. Finally, we identified the recurrent CNVs in different samples and found the highly recurrent CNVs that may have significant roles in FAs.

We also examined fusion and SV variants using the genefusion and tophat‐fusion software, but did not find the fusion or SV variants in these FAs.

### 2.5 Assessment of TMB

For TMB, the number of somatic single nucleotide variations and indels detected on NGS were quantified. Somatic variants were filtered by the Mutect2 FilterMutectCalls module, variants out of gene function region were excluded, but the synonymous variants were retained. Alterations listed as known somatic alterations in COSMIC and known germline alterations in dbSNP, 1000 G, ExAC database were not counted.^34^ To calculate the TMB per megabase, the total number of mutations counted is divided by the size of the coding region of the targeted territory.^35^ We used 58 Mb as the estimate of the exome size.

### 2.6 Statistics

Statistical analyses were carried out using IBM SPSS version 21.0. The difference in means of TMB from germline mutation positive and negative group was assessed with Student's *t* test. Associations between the clinicopathological characteristics and germline mutations were evaluated through Chi‐square test or Fisher exact test. Two‐tailed *P* values <0.05 were considered statistically significant.

## 3 RESULTS

### 3.1 Somatic mutation spectrum in FA of Chinese population

**Figure 1:**
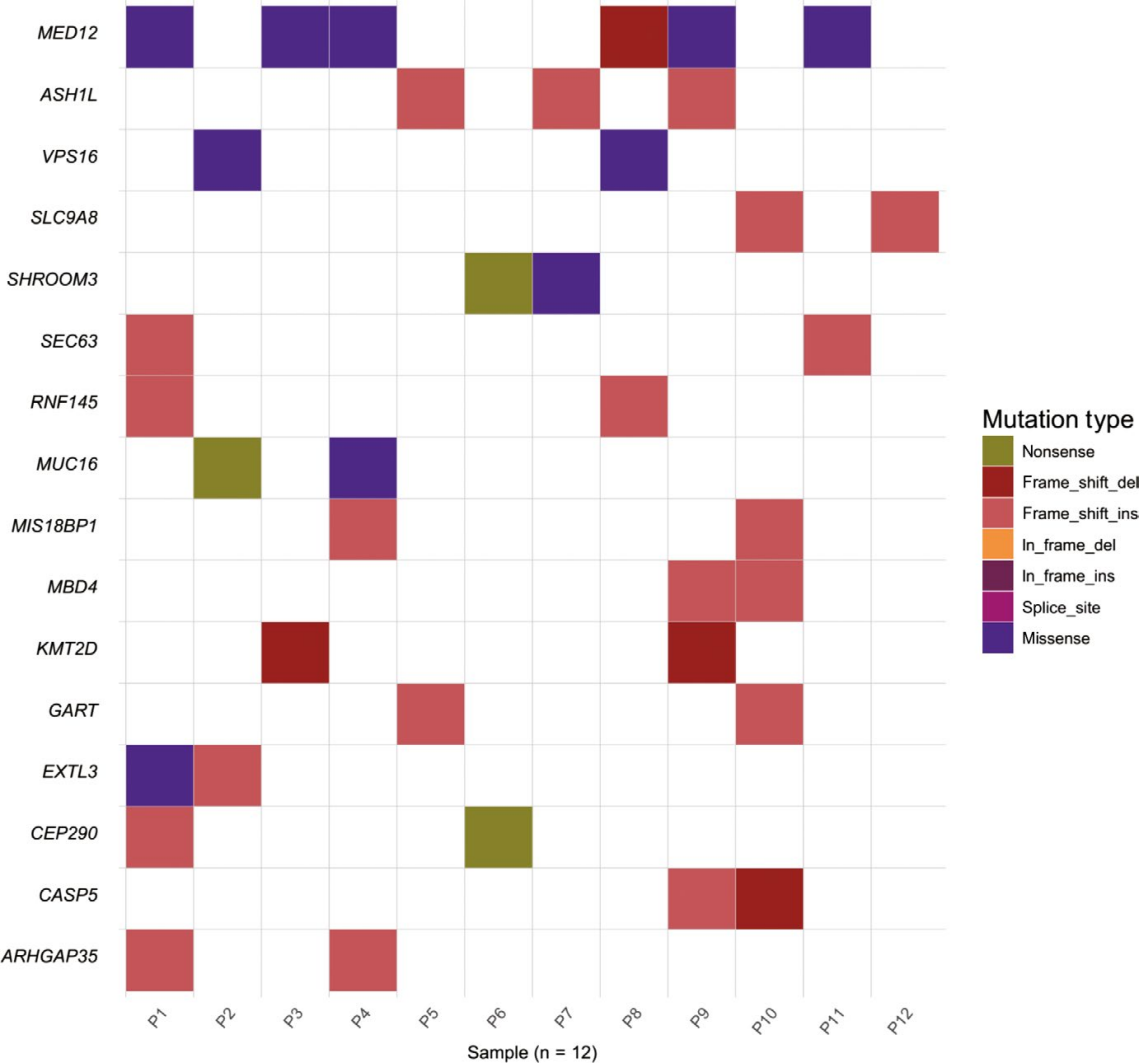
Somatic mutation landscape in fibroadenoma of Chinese Han population. Each column represents 1 sample; genes are reported in rows. Only the genes mutated in at least 2 cases were included. Alteration types are color‐coded according to the legend

To identify genes with recurrent somatic mutations across multiple samples, we subjected 12 pairs of FAs and the corresponding normal tissues to whole exome sequencing. Then we analyzed the coding regions and flanking regions of genes according to the somatic mutation analysis pipeline (Figure S2). One hundred thirty‐seven missense mutations and 198 LoF mutations were identified, but only 16 genes with somatic mutations in more than 1 sample (Figure 1 and Table 1). The mean number of somatic mutations in each sample were 3 with a range of 2‐6. Among the 16 genes, *MED12* was the most common mutant genes that was observed in 6 cases (50%, 6/12). In addition, frameshift mutation, including 4 frameshift‐deletions and 20 frameshift‐insertions, accounted for 65% (24/37) of all somatic mutations, followed by missense mutation (10/37, 27%).

**Table 1:** Summary of mutations of 16 genes in 12 fibroadenoma samples

Gene	Mutation type			Frequency
	Nonsense	Missense	Frameshift	
*MED12*	0	5	1	50.0% (6/12)
*ASH1L*	0	0	3	25.0% (3/12)
*VPS16*	0	2	0	16.7% (2/12)
*SLC9A8*	0	0	2	16.7% (2/12)
*SHROOM3*	1	1	0	16.7% (2/12)
*SEC63*	0	0	2	16.7% (2/12)
*RNF145*	0	0	2	16.7% (2/12)
*MUC16*	1	1	0	16.7% (2/12)
*MIS18BP1*	0	0	2	16.7% (2/12)
*MBD4*	0	0	2	16.7% (2/12)
*KMT2D*	0	0	2	16.7% (2/12)
*GART*	0	0	2	16.7% (2/12)
*EXTL3*	0	1	1	16.7% (2/12)
*CEP290*	1	0	1	16.7% (2/12)
*CASP5*	0	0	2	16.7% (2/12)
*ARHGAP35*	0	0	2	16.7% (2/12)

**Figure 2:**
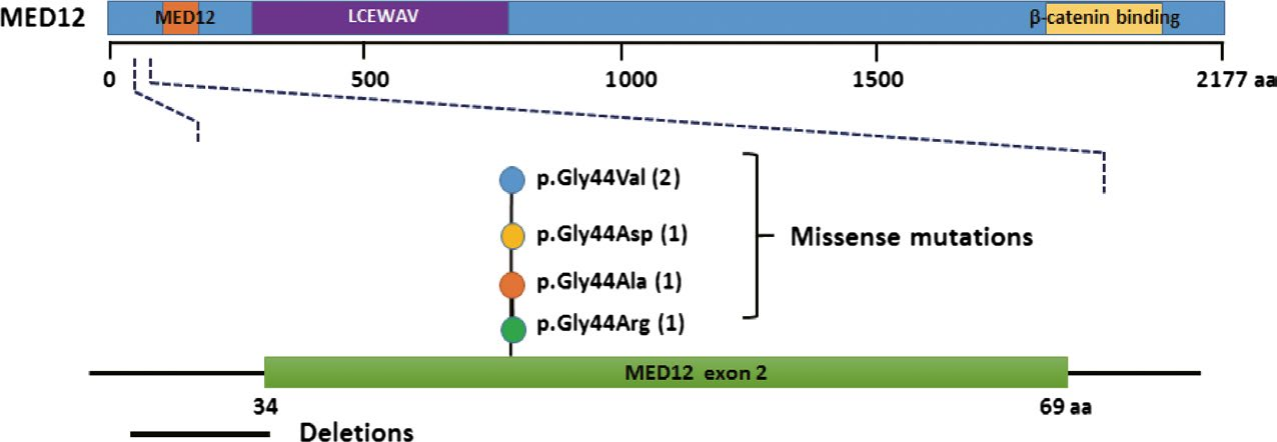
Schematic representation highlights the distribution of *MED12* exon 2 mutations identified in fibroadenomas in this study. *MED12* is shown with high‐confidence Pfam protein domains. A close‐up view of residues in *MED12* exon 2 indicates the location of *MED12* alterations found in this study. The frequency of each alteration is denoted in parentheses after its label. A strong preference for P.Gly44 substitutions can be observed. aa, amino acid

**Figure 3:**
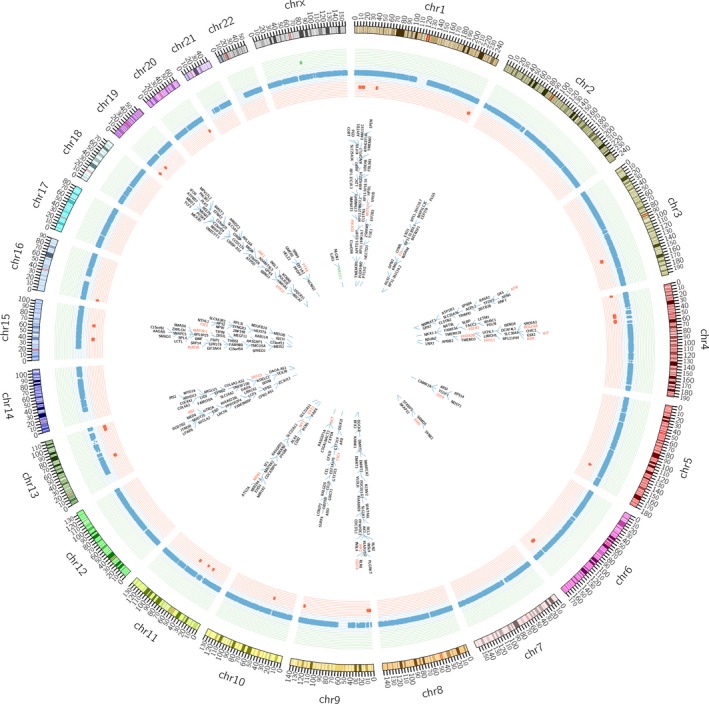
Circos plots for the 12 fibroadenoma samples. The 27 somatic recurrent copy number variants (CNVs) (*n* ≥ 2) were shown in panels (red, deletions; green, duplications). The genes involved CNVs were listed in the interior of the plots. Genes in red were oncogenes; gene in green was *MED12 *gene

As a remarkably high frequency of *MED12* mutation in FAs, we then analyzed mutations of *MED12* in 12 samples independently. A total of 6 *MED12* mutations were identified in the 12 FA samples (50%, 6 of 12) and all of them were located in exon 2 and franking intron 1, which were in accordance with previous studies.^17^, ^18^ None of the FA cases harbored more than 1 mutation. All but 1 of the mutations were missense mutations and restricted in codon 44, a hot‐spot of *MED12* mutations reported in FAs. The remaining 1 was frameshift mutation which was located in the 5′‐coding sequences of exon 2 of *MED12*, resulting in the loss of a splice acceptor (Figure 2).

In addition, we analyzed the somatic CNVs according to the somatic CNV analysis pipeline (Fig S3). A total of 27 recurrent somatic CNVs (26 deletions and 1 duplication) were identified in 12 FA samples (Figure 3). Among these genes, duplication of *MED12* in codon 38 was detected in 6 FA samples (P4, P5, P7, P9, P10, P12). Out of these, 27 oncogenes were involved in the CNVs.

### 3.2 Germline mutations of tumor susceptibility genes in FA of Chinese Han population

To gain insight into the germline mutations of tumor susceptibility genes in 12 FA samples, we analyzed germline mutations that were presented among 151 tumor susceptibility genes according to the analysis pipeline in Figure S4. Germline aberrations revealed by WES analysis in 12 FA samples are summarized in Table 2. A total of 6 missense mutations were identified in 5 samples (P1, P3, P4, P6, P10). All but 1 of the samples harbored only one mutations among 151 tumor susceptibility genes, whereas P1 harbored 2 mutations.

**Table 2:** Summary of variants in 151 tumor susceptibility genes among 12 samples

Gene	cDNA change	Protein change	NM ID	Patient ID	AF
*APC*	c.6680G>T	p.Gly2227Val	NM_000038.5	P1	52.50%
*WRN*	c.104T>C	p.Val35Ala	NM_000553.4	P1	44.44%
*ATM*	c.8246A>T	p.Lys2749Ile	NM_000051.3	P4	50.88%
*ERCC2*	c.691G>A	p.Val231Met	NM_000400.3	P3	54.64%
*FLCN*	c.170G>A	p.Arg57Gln	NM_144997.5	P10	47.50%
*CDK4*	c.776C>T	p.Ser259Leu	NM_000075.3	P6	46.67%

AF, allele frequency.

**Table 3:** Correlation between germline variations in tumor susceptibility genes and TMB in fibroadenoma of Chinese Han population

Sample type	Patient ID	TMB	TMB *P*‐value
Germline pathogenicity variation group	P1	5.6	*P* = 0.0036
	P3	3.96	
	P4	5.46	
	P6	7.55	
	P10	6.00	
Germline no pathogenicity variation group	P2	1.91	
	P5	3.27	
	P7	2.89	
	P8	2.24	
	P9	5.12	
	P11	3.37	
	P12	2.68	

TMB, tumor mutational burden.

To clarify the relationship between germline mutations of tumor susceptibility genes and TMB in FA of Chinese Han population, we divided the patients into 2 groups (patients with and without germline mutations) and calculated the TMB of each sample. Our findings indicated that the TMB of FAs with germline mutations was significantly higher than that without germline mutations (*P* < 0.05) (Table3). Meanwhile, we also analyzed the relationship between germline mutations of tumor susceptibility genes and clinicopathological characteristics of FAs, but no significant differences were found.

## 4 DISCUSSION

As the most common benign tumor of the breast in China, with an approximately twofold increase in relative risk of developing invasive breast carcinoma after 20 years (long‐term risk of breast cancer in women with FA), FA should get more attention from researchers.^1^, ^2^ At present, the genetic abnormalities that underlie FA remain incompletely understood. In this study, we performed a mutational analysis in 12 FAs by means of WES and identified somatic and germline mutation spectrum of FAs in Chinese Han population, which provide insights into molecular pathogenesis of FAs.

Lim WK et al reported that out of the 98 FA samples sequenced, 41 (42%) had point mutations in codon 44 of *MED12* (20 p.Gly44Asp, 12 p.Gly44Ser, 3 p.Gly44Arg, 3 p.Gly44Val, 2 p.Gly44Cys, and 1 p.Gly44Ala).^17^ Tan J et al reported that frequent mutations of *MED12 *were identified in all subtypes of FAs.^19^ In our study, we identified recurrent somatic mutations of *MED12* in 6 cases (6/12, 50%), with 83% of mutations occurring in codon 44 (5/6), emphasizing the importance of these mutations in FA tumorigenesis. Previous studies indicated that *MED12* was found to be recurrently mutated in hormone‐associated cancers, such as prostate cancer, adrenocortical carcinoma, and uterine leiomyomas (ULs).^36^, ^37^ Among these tumors, only FAs and ULs have the nearly identical *MED12* mutation spectrum in both exon location and variant codon preference, suggesting a common underlying molecule mechanism in ULs and FAs that *MED12* exon 2 mutations could be associated with hormonal expression.

Though most of the FAs can be explained by the mutations of *MED12*, the pathogenesis of other FAs without *MED12 *mutations remains unknown, so we speculate that FAs may be heterogeneous. In previous studies, Lim et al identified 41 genes exhibiting 45 confirmed somatic mutations in FAs, almost all the genes mutated in only 1 patient, except *MED12 *which was occurred in 59%(58/98) cases. But the mean coverage of sequencing was 124×.^17^ Tan et al reported that 20 recurrently mutated genes were identified in 100 fibroepithelial tumors (including 21 FAs and 34 benign, 35 borderline and 10 malignant PTs), and the mean coverage of the target genes was 524× (minimum 228×). In 21 FAs, missense mutation was found in *MED12 *(11 cases), *RARA *(2 cases), *FLNA *(1 case), *PCLO *(1 case),* CHD8 *(1 case), and *ROS1 *(1 case).^19^ Lozada et al reported myxoid FAs are different from conventional FAs, which lack *MED12* mutations.^24^ So we speculate that the fewer recurrently mutated genes were reported by Lim et al may attribute to the lower coverage. In this study, we conducted the WES with the mean coverage of 227×, and 15 recurrent genes with somatic mutations were found in addition to *MED12*. Among these genes, *KMT2D* has been reported in a FA case with a frameshift mutation (p.Gln4347fs).^39^ Tan et al reported that loss‐of‐function mutations in *KMT2D* has been observed in PTs, but were rarely present in FAs.^19^ In this study, we identified 2 frameshift mutations (p.Lys1840fs and p.Val3527fs) which have not been reported previously in 2 FA cases (P3 and P9). This finding indicated that recurrent somatic mutations in *KMT2D *might be the shared markers of FAs.

After studying the SNVs and insertion/deletions, we also researched the somatic copy number variations (CNVs) in FAs that also played import roles in tumorigenesis. We detected 27 recurrent somatic CNVs, most of which clustered in chr1, 4, 9, 11, 13, 15, 19 and were deletions. These were different from previous study, in which it was reported that gains rather than losses of DNA fragments were a feature of FAs and the most frequently overrepresented segments clustered in chr5, 10, 13, 18.^40^ What's more, among the CNVs, we found a recurrent *MED12* duplication in 6 cases, indicating the importance of *MED12 *in FAs.

According to the proliferations of epithelial and stromal elements, FAs are divided into the pericanalicular and intracanalicular patterns. In order to find out the pathogenic mechanism of different types of FAs, we compared the somatic mutations between them, but no significant difference of genetic spectrum was found, maybe attributing to the small number of samples.

Germline mutations of the FAs were rarely studied. Previous reports indicated that multiple and/or bilateral FAs were associated with a family history,^13^ the myxoid FAs were also associated with Carney Complex.^24^ So we analyzed germline mutations of tumor susceptibility genes, and identified 6 mutations in 6 genes, but no difference was found in clinicopathological characteristics between cases with germline mutations and without germline mutations, which might be attributed to the small number of cases. TMB of FAs with germline mutations was significantly higher than that without germline mutations, indicating that persons with germline mutations in tumor susceptibility genes might have increased incidence of somatic mutations.

This study showed the genetic landscapes of FAs in Chinese Han population, which expand the genetic spectrum of FAs, and provided important clinical implications. However, the genetic landscapes of FAs may be not comprehensive due to the small number of samples in this study. More cases should be used in the future to clarify the genetic basis of FAs and verify the roles of the novel genes with somatic mutations identified in this study.

## CONFLICT OF INTEREST

The authors declare that they have no conflict of interest.
