# Supplementary material for: The genomic mutation spectrums of breast fibroadenomas in Chinese population by whole exome sequencing analysis
Source: Cancer Med. 2019 Mar 9;8(5):2372–9. doi: 10.1002/cam4.2081 (PMC6536971; doi:10.1002/cam4.2081)
Supplement: Supplementary file 1 [file CAM4-8-2372-s001.pdf]

Table S1 Clinicopathological characteristics of FA

Patient ID#	Age	histological type	epithelial hyperplasia	ADH/UDH	single/ multiple	location
P1	39	Intracanalicular	mild-moderate	+	multiple	unilateral
P2	24	Intracanalicular	NA	-	multiple	bilateral
P3	42	Intracanalicular	mild	-	multiple	bilateral
P4	22	Intracanalicular	mild-moderate	+	multiple	bilateral
P5	16	Juvenile adenoma	varying degrees	+	single	unilateral
P6	45	complex	active	+	single	unilateral
P7	20	Pericanalicular	focal epithelium hyperplasia	-	multiple	unilateral
P8	35	Intracanalicular	active	+	single	unilateral
P9	36	Intracanalicular	mild-moderate	+	single	unilateral
P10	30	Intracanalicular	active	+	single	unilateral
P11	18	Pericanalicular	active	+	multiple	bilateral
P12	43	Intracanalicular	mild	+	multiple	bilateral

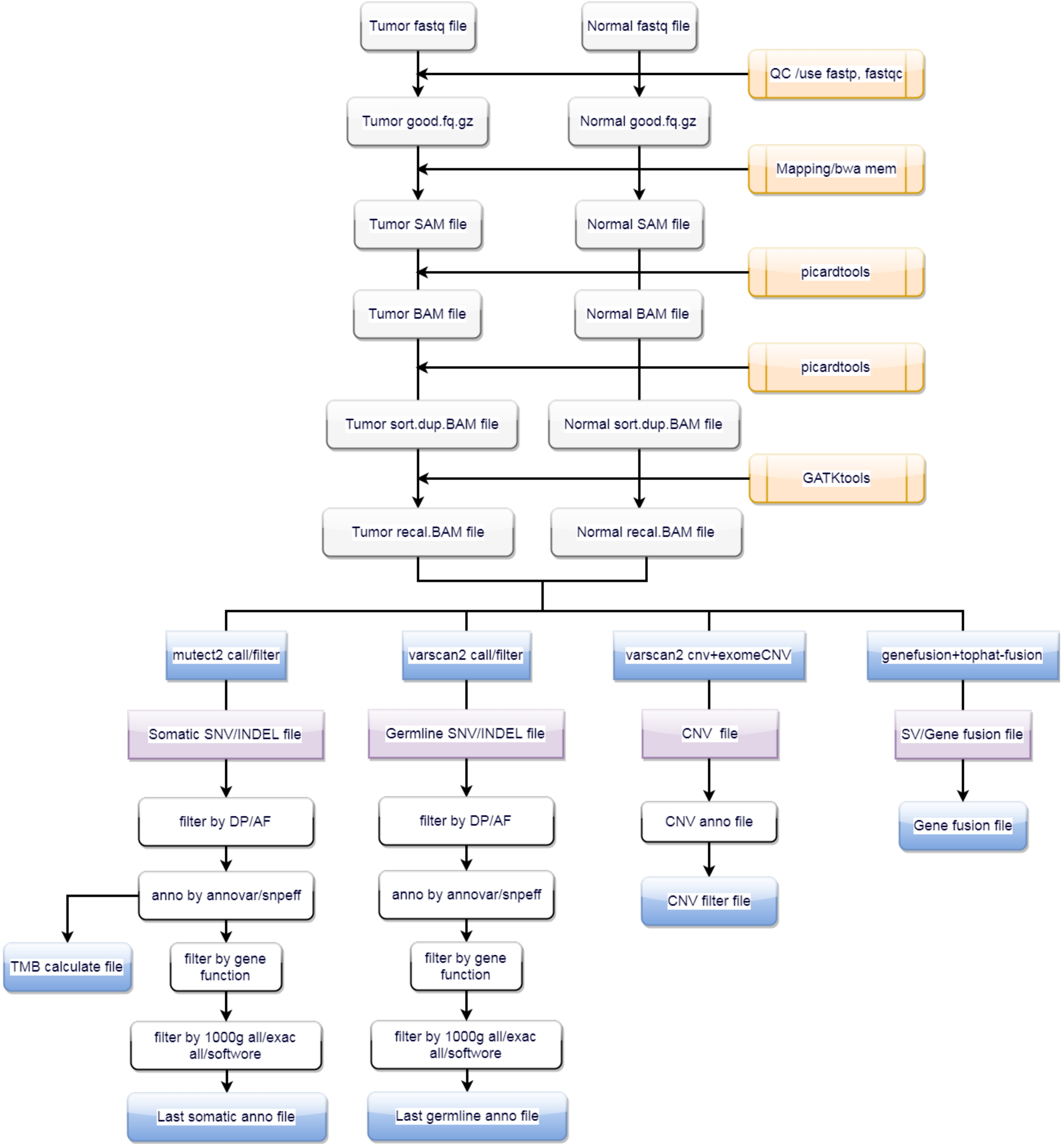

Fig S1 The bioinformatic analysis pipeline of the 12 samples.

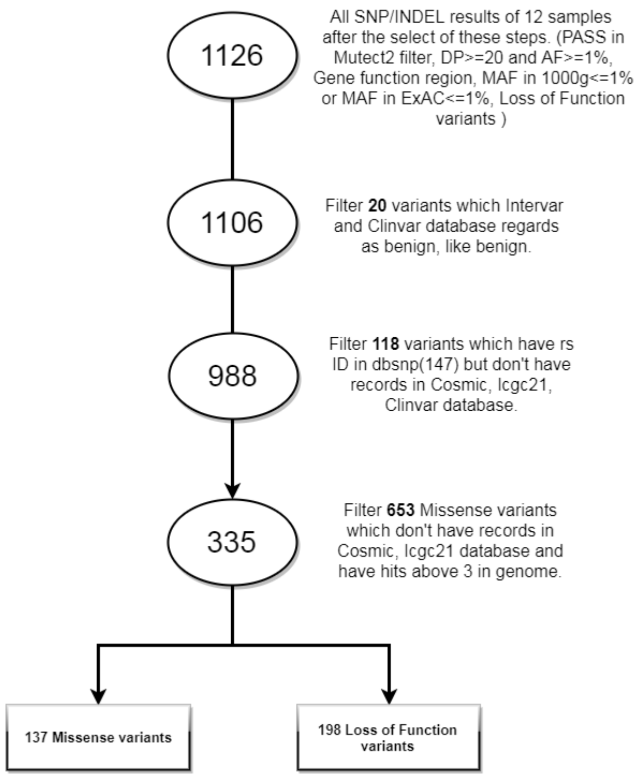

Fig S2 The bioinformatic analysis pipeline for somatic mutation.

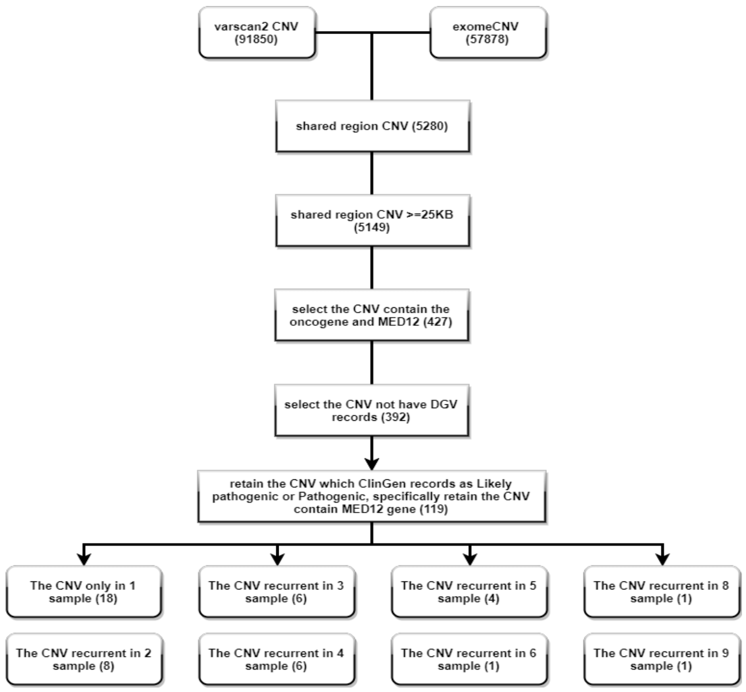

Fig S3 The bioinformatic analyse pipline for somatic CNV.

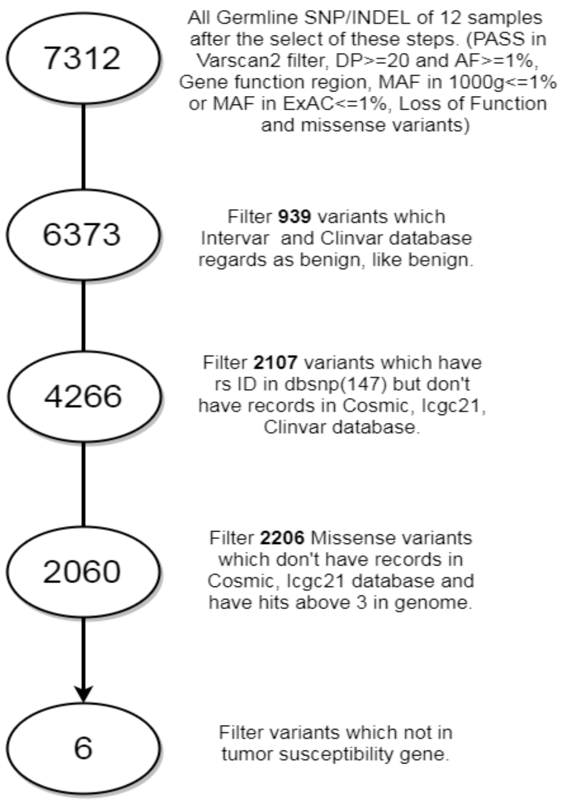

Fig S4 The bioinformatic analysis pipeline for germline mutations of tumor susceptibility genes.
